# Variability of DNA Repair and Oxidative Stress Genes Associated with Worst Pain in Breast Cancer Survivors on Aromatase Inhibitors

**DOI:** 10.3390/genes14112031

**Published:** 2023-10-31

**Authors:** Monica A. Wagner, Theresa A. Koleck, Alex Conway, Catherine M. Bender, Yvette P. Conley

**Affiliations:** 1 Frances Payne Bolton School of Nursing, Case Western Reserve University, Cleveland, OH 44106, USA; maw269@case.edu; 2 School of Nursing, University of Pittsburgh, Pittsburgh, PA 15260, USA

**Keywords:** pain, genetics, oxidative stress, DNA repair, breast neoplasms

## Abstract

Pain is a problem affecting women with breast cancer (HR+BrCa) receiving aromatase inhibitor (AI) therapy. We investigated the relationship between single-nucleotide polymorphisms (SNPs) in DNA repair and oxidative stress genes and perceived worst pain after 6 months of AI therapy. We explored 39 SNPs in genes involved in DNA repair (*ERCC2*, *ERCC3*, *ERCC5*, and *PARP1*) and oxidative stress (*CAT*, *GPX1*, *SEPP1*, *SOD1*, and *SOD2*) in women with HR+BrCa receiving adjuvant therapy (AI ± chemotherapy; *n* = 138). Pain was assessed via the Brief Pain Inventory. Hurdle regression was used to evaluate the relationship between each associated allele and (1) the probability of pain and (2) the severity of worst pain. *ERCC2*rs50872 and *ERCC5*rs11069498 were associated with the probability of pain and had a significant genetic risk score (GRS) model (*p* = 0.003). *ERCC2*rs50872, *ERCC5*rs11069498, *ERCC5*rs4771436, *ERCC5*rs4150360, *PARP1*rs3219058, and *SEPP1*rs230819 were associated with the severity of worst pain, with a significant GRS model (conditional mean estimate = 0.45; 95% CI = 0.29, 0.60; *p* < 0.001). These results suggest DNA repair and oxidative stress pathways may play a role in the probability of pain and the severity of worst pain. As healthcare delivery moves towards the model of precision healthcare, nurses may, in the future, be able to use these results to tailor patient care based on GRS.

## 1. Introduction

Advances in screening and treatment for cancer have led to increased survival for those diagnosed with malignancies. Currently, the overall five-year survival rate for women diagnosed with breast cancer is 91% [1]. The most common early-stage breast cancer in postmenopausal women is hormone-receptor-positive breast cancer (HR+BrCa) [2,3]. For these women, endocrine therapy with an aromatase inhibitor (AI) applied for a period of 5 to 10 years is the standard of care [3]. AIs are designed to prevent cancer recurrence via systematically inhibiting the aromatase enzyme, thereby reducing the production of estrogen by nearly 90% [4,5,6,7]. Unfortunately, as the number of women surviving HR+BrCa increases, so too does the number who experience cancer and cancer-treatment-related symptoms. Pain is a significant problem in up to 60% of women diagnosed with HR+BrCa receiving AI therapy [5]. The most-reported pain among these women is musculoskeletal pain affecting the hands, wrists, knees, and ankles. Pain typically emerges soon after AI therapy initiation, with the median time for pain to emerge being six weeks after therapy initiation and peak pain experience occurring at six months [5,6]. As a result of their pain, these women experience deteriorations in functional status and present decreased adherence to therapy and increased utilization of healthcare resources [5,6]. Despite tremendous research efforts, little is known about the molecular mechanisms underlying pain related to AI therapy in women with HR+BrCa. Variability in DNA repair and oxidative stress genes are implicated in both the development of pain [8,9] and breast cancer [10,11,12]. But there is a dearth of information on the relationship between polymorphisms in DNA repair and oxidative stress genes and the development of pain in this population.

Women with HR+BrCa experience disruptions in both oxidative stress and DNA repair mechanisms. Oxidative stress is involved in the activation of numerous signaling pathways, including the processes of tumor cell migration and proliferation [13]. Many cancer treatments rely upon the activation of the oxidative stress pathway to kill tumor cells [13,14,15]. Different HR+BrCa stages can produce differing profiles of oxidative stress [16]. Levels of oxidative stress can also vary based on the presence of a tumor, the progression of tumor growth, lymph node involvement, and the type of cancer treatment [17,18,19]. Oxidative stress has been linked to tumor receptor status (estrogen/progesterone) and may persist for up to six months after tumor removal [13,20,21]. DNA repair enzymes help maintain stability in the genome, and malfunctions in DNA repair pathways are linked to the progression and development of breast cancer [22,23]. DNA repair is influenced by both estrogen receptor and human epidermal growth factor receptor 2 (*HER2*) status, and it has been suggested that women with HR+BrCa should be stratified based on DNA repair enzyme phenotypes [24].

The relationship between polymorphisms in DNA repair and oxidative stress genes and pain development is not as well defined, but there is evidence suggesting that polymorphisms in DNA repair genes are associated with abdominal/pelvic pain development [25]. The literature also suggests a relationship between oxidative stress genes and the development of pain syndromes such as fibromyalgia [26], complex regional pain syndrome [27], low back pain [28], and joint diseases such as rheumatoid arthritis and osteoarthritis [29]. What is not known is whether an individual susceptible to cancer is also susceptible to cancer-related symptoms, such as pain. To advance knowledge in this area of study as well as nursing science, we explored the relationships between polymorphisms in DNA repair (*ERCC2*, *ERCC3*, *ERCC5*, and *PARP1*) and oxidative stress (*CAT*, *GPX1*, *SEPP1*, *SOD1*, and *SOD2*) genes and perceived worst pain in postmenopausal women with early-stage HR+BrCa after six months of AI therapy. We calculated multi-gene, multi-polymorphism genetic risk scores (GRSs) to assess the collective effect of multiple, individually significant polymorphisms on pain in women with HR+BrCa.

## 2. Materials and Method

### 2.1. Study Participants

The current study capitalizes on clinical, symptom, and genotype data from participants initially recruited in a larger study investigating the effect of adjuvant AI therapy (i.e., anastrozole) ± chemotherapy on changes in cognitive function prior to and throughout an AI therapy regimen [30,31]. The sample for the current analysis consisted of 138 women diagnosed with early-stage (0—IIIa) HR+BrCa (AI + chemotherapy, *n* = 55; AI alone, *n* = 83). Participants were no more than 75 years old, able to speak and read English, had completed at least 8 years of education, and had no diagnoses of psychiatric illness, neurologic disease/trauma, or history of cancer at the time of enrollment. Women with breast cancer were diagnosed based on the TNM (tumor, nodes, and metastasis) classification of malignant tumors, with no clinical evidence of distant metastases [32]. Informed consent was obtained from all participants. This study was approved by the University of Pittsburgh Institutional Review Board.

### 2.2. Evaluation of Perceived Worst Pain

For this study, we used perceived pain scores collected via the Brief Pain Inventory (BPI) [33] after six months of continuous AI therapy. The BPI is an 11-item self-report measure of pain with four items that measure pain severity and seven items that measure pain interference, with higher scores signifying greater pain. We chose the item that asks participants to rate their “worst” pain from 0 (no pain) to 10 (pain as intense as can be imagined) to measure presence of pain and pain severity as this item has shown to be valid and reliable and is frequently used in clinical trials [34].

### 2.3. Single-Nucleotide Polymorphism (SNP) Selection and Genotyping 

The complete procedure for SNP selection was previously described in [31]. Briefly, five candidate oxidative stress genes and four candidate DNA repair genes were identified from the literature [35,36,37,38,39,40,41,42,43,44]. The first choice corresponded to functional polymorphisms, but when a functional polymorphism was not available or did not represent variability in a gene, tagging SNPs were selected using the Phase III HapMap database. A total of 39 SNPs were used (Table 1).

The procedure used for genetic sample collection, extraction, and genotype data collection has also been previously described [31]. Briefly, either saliva or whole blood was obtained from participants. Oragene DNA collection kits were used to extract DNA from saliva, and a simple salting-out method was used to extract DNA from peripheral blood lymphocytes. A 1X TE buffer at −20 °C was used to store extracted DNA. A TaqMan allele discrimination platform (Thermo Fisher Scientific Inc., Waltham, MA, USA) or an iPLEX MassARRAY multiplex assay platform (sequenom, San Diego, CA, USA) was used to determine genotypes. Genotypes were double-called by persons blinded to the subject phenotypes. Any discrepancies were dealt with by reviewing raw data or re-genotyping. Participant genotypes were classified for data analysis based on the presence (i.e., homozygous variant genotype plus heterozygous genotype) or absence (i.e., wildtype genotype) of the minor allele. 

### 2.4. Covariate Assessment 

Bivariate analyses were performed to identify and adjust the potential covariate effects of age, years of education, and levels of depressive symptoms, anxiety, and fatigue on pain. These covariates were chosen based on the influence of cognitive processes, depression, anxiety, and fatigue on perceived pain [45,46]. Pearson’s correlation was used to analyze continuous variables, which included age, education, depression (Beck Depression Inventory II) [47], anxiety (Profile of Mood States Tension—Anxiety subscale) [48], and fatigue (Profile of Mood States Fatigue—Inertia subscale) [48]. Pearson’s Chi-Square was used to evaluate cancer stage.

### 2.5. Statistical Analysis 

We conducted statistical analyses using Stata SE 17.0 and IBM SPSS Statistics Version 27 (IBM Corp., Armonk, NY, USA). To account for biological differences in breast cancer tumors, we classified women by prescribed treatment regimen as a surrogate for disease characteristics. The groups were AI + chemotherapy and AI alone.

Because of the zero-inflated distribution of worst pain scores (i.e., ~50% of worst pain scores were 0), we used a Cragg two-equation hurdle regression to explore the effect of genotype on “worst” pain after six months of continuous AI therapy while controlling for age and symptoms of anxiety, depression, and fatigue in each equation. We fit a linear hurdle model combining a selection model (any pain vs. no pain) and an outcome model (pain score of 1 or greater) with a lower limit of 0. Both models included all predictors. We compared prescribed treatment groups (AI + chemotherapy and AI alone) and possession of one or more minor alleles (homozygous variant genotype plus heterozygous genotype) to the reference (wildtype) genotype. We used regression coefficients, probabilities, and significance tests from the selection model to assess the average marginal effect of GRS on probability of pain. Then, we used regression coefficients, conditional (i.e., controlled for other variables in the model) mean estimates (CMEs) of worst pain score, and significance tests from the outcome model to investigate the influence of genotype on worst pain severity in women with a pain score of 1 or greater. 

We calculated the GRS for each participant using SPSS. The GRS measures the collective effect of DNA repair and oxidative stress polymorphisms on worst pain ratings. We included SNP alleles associated (*p* ≤ 0.05) with worst pain in the individual main effect only and/or interaction effect models in the GRS calculation for both the selection and outcome models. We used a weighted method to assign greater risk/protection to alleles with stronger associations. Unstandardized regression β-coefficients from individual SNP models were multiplied by 0 (absence) or 1 (presence) based on a participant’s genotype and membership in the AI + chemotherapy treatment group and then totaled. 

The equation for the selection GRS, where “Group” represents AI + chemotherapy, is as follows:

(0.645 × ERCC2rs50872 − T) + (−0.601 × ERCC2rs50872 − T × Group) + (0.277 × ERCC5rs11069498 − G) + (−1.347 × ERCC5rs11069498 − G × Group)

And the equation for the outcome GRS is as follows:

(−1.522 × ERCC2rs50872 − T) + (1.484 × ERCC2rs50872 − T × Group) + (−1.313 × ERCC5rs11069498 − G) + (1.396 × ERCC5rs11069498 − G × Group) + (−1.128 × ERCC5rs4150360 − C) + (2.188 × ERCC5rs4150360 − C × Group) + (−1.344 × ERCC5rs4771436 − G) + (1.065 × ERCC5rs4771436 − G × Group) + (1.041 × PARP1rs3219058 − A) + (−3.175 × PARP1rs3219058 − A × Group) + (−0.789 × SEPP1rs230819 − A) + (3.349 × SEPP1rs230819 − A × Group).

As an example, we calculated the outcome model GRS for a participant prescribed AI + chemotherapy and possessing the minor alleles for ERCC5rs11069498 and SEPP1rs230819 as follows:

GRS = (−1.522 × 0) + (1.484 × 0 × 1) + (−1.313 × 1) + (1.396 × 1 × 1) + (−1.128 × 0) + (2.188 × 0 × 1) + (−1.344 × 0) + (1.065 × 0 × 1) + (1.041 × 0) + (−3.175 × 0 × 1) + (−0.789 × 1) + (3.349 × 1 × 1) = 2.643

A higher GRS indicates greater genetic risk of pain presence or higher severity of worst pain, while a lower GRS indicates greater genetic protection. Participants missing genetic data necessary for completion of a GRS calculation were not included in the GRS analyses.

### 2.6. Pain Location Data

After calculating the GRS, we performed a post hoc descriptive analysis of pain location to determine where participants were experiencing pain and if the areas of pain shifted over time. Pain location data were collected in accordance with item number two of the BPI [33], where participants use an “X” to mark on a diagram the areas of their body that hurt “today”. Data were compiled by summing the number of “Xs” in the areas of the breast/axilla to represent persistent pain experienced by 25% to 60% of the women following breast cancer surgery [49,50] and in more distal areas (e.g., knee, lower back, fingers, and thumb) to represent AI-induced joint pain [51,52]. Results indicate the number of “Xs” counted in each area. 

## 3. Results

Each SNP was tested for Hardy–Weinberg equilibrium using a Chi square goodness-of-fit test. In the sample of 138 women, seven SNPs, *CAT*rs2179625 (X^2^ = 4.273, *p* = 0.039), *ERCC2*rs3916874 (X^2^ = 387.731, *p* < 0.001), *ERCC5*rs4150360 (X^2^ = 4.223, *p* = 0.040), *PARP1*rs1136410 (X^2^ = 14.240, *p* < 0.001), *SEPP1*rs28919892 (X^2^ = 5.912, *p* = 0.015), *GPX1*rs1050450 (X^2^ = 189.036, *p* < 0.001), and *SOD1*rs1041740 (X^2^ = 7.172, *p* = 0.007), were not in Hardy–Weinberg equilibrium. The deviation seen in these SNPs is most probably attributable to the lack of random sampling of participants, as this was a cohort of women with HR+BrCa; therefore, all SNPs were included in the final analysis.

### 3.1. Participant Characteristics

Pain and covariate data were available for all 138 participants. The baseline characteristics reveal that the cohorts had statistical, but not clinically meaningful, differences in age (*p* < 0.001), with women receiving AI alone being slightly older (Table 2). There was no significant difference in worst pain severity between women who received AI + chemotherapy and those who received AI alone (Table 2).

### 3.2. Influence of Co-Occurring Symptoms

There were no significant differences in levels of depression or fatigue between women who received AI alone and women who received AI + chemotherapy. However, there was a significant difference between the treatment groups in the level of reported anxiety. At baseline, women who received AI + chemotherapy reported a higher mean level of anxiety compared to women who received AI alone (*p* = 0.005; Table 2).

### 3.3. Polymorphisms Included in GRS for Determining Probability of Having Any Pain

The SNPs *ERCC2*rs50872 and *ERCC5*rs11069498 were both significant and included in the GRS calculation (Table 3). Both SNPs were significant as main effects, with no treatment interaction meaning that both SNPs are associated with the probability of pain regardless of treatment type.

GRS was significant for predicting the probability of pain in this study (*p* = 0.003, Table 3). The average marginal effect of GRS on the probability of pain was 27.72 (95% CI = 9.24, 46.21). This means that for every unit increase in GRS, there was a 27.72% increase in the probability of pain. Fatigue was a significant predictor of the probability of experiencing pain, with a 4.10% average increase per unit increase in fatigue (*p* < 0.001; 95% CI = 2.18%, 6.09%). Treatment was also a significant predictor of the probability of experiencing pain, with a 38.06% average increase for participants who received AI + chemotherapy (*p* = 0.008; 95% CI = 9.80%, 66.32%).

### 3.4. Polymorphisms Included in the GRS for Determining Severity of Worst Pain

The SNPs *ERCC2*rs50872, *ERCC5*rs11069498, and *ERCC5*rs4771436 were all significant as main effects with no treatment interaction (Table 4). The SNPs *ERCC5*rs4150360, *PARP1*rs3219058, and *SEPP1*rs230819 were all significant as treatment interaction effects without a main effect, meaning that the significance of each SNP depended on the treatment (AI + chemotherapy). All six SNPs, both main and interaction effects, were included in the GRS calculation.

After six months of AI therapy, GRS was a significant predictor for severity of worst pain (*p* < 0.001; CME = 0.45; 95% CI = 0.29, 0.60). (Table 4). This means that a higher GRS indicates a greater risk for increased severity of worst pain.

### 3.5. Post Hoc Reported Pain Location Differences at Significant Timepoints

The women with HR+BrCa reported more pain in the breast/axilla region prior to AI therapy than after six months of AI therapy. After six months of AI therapy, the women observed reported pain in more distal areas (e.g., knee, lower back, fingers, and thumbs). The number of breast cancer patients with no pain increased at 6 months; this could have been due to women with more severe pain stopping AI treatment and being lost to attrition (Table 5). 

## 4. Discussion

The findings from this study suggest that genetic variation in DNA repair and oxidative stress pathways may play a role in the probability of pain development and the severity of worst pain after six months of AI therapy. We found that variations in the *ERCC2*, *ERCC5*, *PARP1*, and *SEPP1* genes showed a significant association with the probability of pain and the severity of worst pain in this population. To the best of our knowledge, this is the first study to explore the relationship between polymorphisms in DNA repair and oxidative stress genes and pain in women with HR+BrCa. 

We cannot definitively state that the pain experienced by women in this study was solely due to AI therapy, as the women might still be experiencing pain related to breast cancer surgery. However, the 6-month timepoint was chosen to decrease the chances that pain would be related to surgery. Nearly 25% of women with breast cancer experience substantial levels of breast pain in the first six months following breast cancer surgery [50]; up to 50% of these women may develop chronic breast pain [53]. The area around the breast is the most frequently reported site of pain post-breast cancer surgery, followed by the axilla, arm, and the side of the body [49,53]. At 6 months, pain was reported by women with breast cancer in more distal areas. This is in concordance with previous research findings that symptoms of AI-induced musculoskeletal pain emerge at around 2 to 3 months and are most severe at 6 months [51,52,54]. Breast cancer tumors are heterogenous. To account for this difference in disease characteristics, participants with breast cancer were classified by their prescribed treatment group. In this study, there was no difference in severity of worst pain or location between the women who received AI + chemotherapy and the women who received AI alone. This finding indicates that the presence of pain or the severity of worst pain after six months of AI treatment are not related to the biology of breast cancer. 

Although breast/axilla pain was the most frequently reported pain location prior to AI therapy initiation, there were still many women with distal pain at the same timepoint. Because pain data are subjective and no objective measures were used, it is difficult to distinguish the pain types experienced. Based on the literature, we concluded that breast/axilla pain was attributed to breast cancer surgery, as this pain was experienced by nearly 25% of the women with breast cancer [49,53,55]. On the other hand, it was difficult to discern the source of reported distal pain, as it could be AI-induced musculoskeletal pain, but it could also be more general pain experienced by postmenopausal women due to estrogen loss or the presence of comorbidities [51,56]. 

There was a difference in baseline levels of anxiety between the women who received AI+ chemotherapy and those who received AI alone, with the women who received the combination treatment reporting higher levels of anxiety. This increased anxiety could be related to having a more biologically complex diagnosis or having to receive a more robust treatment regime [57]. Also, there were more women with more advanced stages of cancer in the combination group, as would be expected.

Two SNPs in DNA repair genes, *ERCC2*rs50872 and *ERCC5*rs11069498, but none of the oxidative stress genes were significant for the average marginal effect of the probability of pain (Table 3). This finding is notable considering decreased estrogen is involved in the expression of antioxidant genes and is a risk factor for oxidative stress [58]. Breast cancer cells are susceptible to oxidative damage and have high levels of oxidative stress that may vary depending on the presence and progression of the breast cancer tumor and the different cancer treatments applied, including chemotherapy and surgery [13]. A possible reason for our finding is that oxidative stress leads to DNA damage and the need for DNA repair [59]. Therefore, it could be that we see oxidative stress genes having a greater impact on whether someone has pain prior to the 6-month timepoint, and DNA repair genes later, when DNA repair enzymes are more active in repairing damage. 

Not only is the GRS developed in this project a predictor of the probability of pain, but our findings also suggest that fatigue and treatment type are also predictors. This finding is not surprising, as fatigue is commonly experienced in women with breast cancer and often co-occurs with pain [60,61,62]. Our group has also found that the polymorphisms used for this study may also be associated with cancer-related fatigue [63]. Receiving AI + chemotherapy also increased the probability of pain by ~38%. Women who received AI + chemotherapy in our study had significantly different levels of anxiety compared to the women who were only prescribed AI therapy. This anxiety could be due in part to the fact that the women were receiving chemotherapy. During the first six months of chemotherapy treatment, women with breast cancer show increased levels of anxiety that are in part related to the development of chemotherapy-induced peripheral neuropathy, of which pain is a major symptom [64,65]. Another reason for increased anxiety could simply be the biology of the disease and the fact that women who receive both AI and chemotherapy generally have a more advanced diagnosis [3].

The GRSs calculated in this study were also significant with respect to predicting the severity of worst pain. The severity of worst pain was influenced by SNPs in three DNA repair genes (*ERCC2*rs50872, *ERCC5*rs11069498, *ERCC5*rs4150360, *ERCC5*rs4771436, and *PARP1*rs3219058) and one oxidative stress gene (*SEPP1*rs230819). Two of the SNPs in the DNA repair genes (*ERCC2*rs50872 and *ERCC5*rs11069498) were also significant in terms of predicting the probability of pain, indicating that these SNPs may be globally important in the development of pain. The *ERCC2* and *ERCC5* genes are both involved in nucleotide excision repair, and defects in these genes can lead to a variety of disorders that involve skin sensitivity (xeroderma pigmentosum and Cockayne syndrome) [66]. Allodynia, a condition where a stimulus that normally does not evoke pain (such as a light touch) is painful, is a hallmark sign of neuropathic pain. A classic example of allodynia is the pain felt by clothing rubbing up against freshly sunburnt skin. It could be that variations in genes of the DNA repair pathway are involved in the development of certain painful conditions. Further research is needed to explore the relationship between these genes and pain. 

Seven out of the thirty-nine SNPs included in this analysis were not in Hardy–Weinberg equilibrium. Three of the SNPs (*ERCC2*rs3916874, *ERCC5*rs4150360, and *PARP1*rs1136410) are in DNA repair genes, while the other four SNPs (*CAT*rs2179625, *SEPP1*rs28919892, *GPX1*rs1050450, and *SOD1*rs1041740) are in oxidative stress genes. It is not surprising that not all the SNPs were in Hardy–Weinberg equilibrium, as our sample size was relatively small and restricted to women diagnosed with early-stage HR+BrCa. We would expect that in a larger sample not restricted to women with breast cancer, all SNPs would be in Hardy–Weinberg equilibrium.

In the current study, we found genes and SNPs significant for the probability of pain and pain severity. Although only two SNPS from two genes were significant for probability of pain, both SNPs were also significant for the severity of worst pain. There could be a variety of reasons why there are only two SNPs in the selection model [67]. The most likely reason is that the sample sizes were too small to detect additional SNPs. As the parent study was designed to explore differences in cognitive function, pain was not an original main outcome; therefore, women who stopped receiving AI therapy were dropped from the study, and no further data were obtained from these women. This could have biased the results of the current study, as of the 20% to 30% of women who discontinue AI therapy, 75% cite pain as the major reason [5,6]. 

### 4.1. Implications for Nursing Science and Nursing Practice

The results of the current study have implications for both nursing science and nursing practice. Nurse scientists are leaders in symptom science research, using patients’ clinical, demographic, social, behavioral, and omic information to inform the characterization of individual symptom experiences, help guide self-management, and individualize interventions to alleviate symptoms. As healthcare delivery moves towards the model of precision healthcare, the results of the current study, if replicated, can be used to tailor patient care based on a patient’s GRS. Patients with higher GRSs, who are at a higher risk of severe pain development, can receive (1) guidance regarding their risk of pain development and (2) be provided with options for interventions (pharmacological or non-pharmacological) that may help prevent/reduce the severity of pain. 

### 4.2. Limitations and Future Directions

In this exploratory analysis, we focused on polymorphisms that had previously been used in a study designed to examine cognitive function; therefore, we might not have evaluated variability in all genes in the DNA repair and oxidative stress pathways important with regard to the variability of perceived worst pain. This study’s small sample size limited our ability to use all three genotypes to conduct the genetic analyses, so, instead, we collapsed genotypes into two categories, limiting our ability to determine the gene–dose effects. Moreover, our sample consisted of postmenopausal women with early-stage HR+BrCa who reported being primarily non-Hispanic. Therefore, our ability to generalize the results is limited. A larger, more diverse sample is required to explore these variables in a meaningful manner to enhance generalizability. As the parent study was designed to examine cognitive function in women prescribed AI therapy, data collection was stopped for those participants who stopped taking AI therapy, limiting the ability to follow these women beyond their withdrawal from the study. 

## Figures and Tables

**Table 1:** Candidate DNA repair and oxidative stress genes with analyzed SNPs.

DNA Repair Genes		Oxidative Stress Genes	
**Excision Repair Cross-Complementation Group 2 (*ERCC2*)**	**Excision Repair Cross-Complementation Group 5 (*ERCC5*)**	**Catalase (*CAT*)**	**Selenoprotein P, Plasma 1 (*SEPP1*)**
rs13181	rs11069498	rs1001179 ^a^	rs230819
rs1799786	rs2296147	rs10488736	rs28919892
rs1799787	rs2296148 ^a^	rs2179625	rs3877899 ^a^
rs238406	rs4150355	rs511895	
rs238416	rs4150360	rs525938	
rs3916874	rs4771436	rs566979	Superoxide Dismutase 1, Soluble (*SOD1*)
rs50871	rs751402	rs769214 ^a^	rs1041740
rs50872	rs873601		
**Excision Repair Cross-Complementation Group 3 (*ERCC3*)**	**Poly (ADP-ribose) Polymerase 1 (*PARP1*)**	**Glutathione Peroxidase 1 (*GPX1*)**	**Superoxide Dismutase 2, Mitochondrial, (*SOD2*)**
rs2134794	rs1136410 ^a^	rs1050450	rs4880 ^a^
rs4150402	rs2271347		rs5746136
rs4150407	rs3219058		rs8031
rs4150477	rs3219090		

^a^ *Functional polymorphism*.

**Table 2:** Participant characteristics: differences between the treatment types. Significance tests noted by *.

Characteristic	AI Alone (*n* = 83)	AI + Chemotherapy (*n* = 55)	F or X^2^ Test Statistic *p*-Value
**Age (mean years ± SD)**	62.47 ± 5.96	58.76 ± 5.47	<0.001 *
**Education (mean years ± SD)**	14.95 ± 3.06	15.67 ± 2.78	0.162
**Depression (BDI-II, mean score ± SD)**	4.60 ± 4.65	5.24 ± 4.61	0.428
**Race**			
**White (count, %)**	81 (97.59)	52 (94.55)	0.387
**Black (count, %)**	1 (1.20)	3 (5.45)	-
**Native American (count, %)**	1 (1.20)	0	-
**Ethnicity, Non-Hispanic/Non-Latino**	83 (100)	55 (100)	-
**Cancer Stage**			
**Stage 0 (count, %)**	1 (1.20)	0	-
**Stage I (count, %)**	68 (81.93)	25 (45.45)	-
**Stage IIa (count, %)**	12 (14.46)	19 (34.54)	-
**Stage IIb (count, %)**	2 (2.41)	5 (9.09)	-
**Stage IIIa (count, %)**	0	6 (10.90)	-
**Anxiety (POMS tension-anxiety subscale, score ± SD)**	6.97 ± 4.65	9.61 ± 6.14	0.005 *
**Fatigue (POMS fatigue-inertia subscale, mean score ± SD)**	5.84 ± 6.35	5.11 ± 5.33	0.481
**Married status (count (%))**	54 (65.1)	38 (69.1)	0.759
**Number of children (mean ± SD)**	2.05 ± 1.39	1.75 ± 1.22	0.191
**6-Month Worst Pain (mean score ± SD)**	3.95 ± 3.32	4.18 ± 3.52	0.705

**Table 3:** Impact of GRS on the probability of pain: results of the hurdle regression for the selection model.

Average Marginal Effect on Probability Of Pain (95% CI)	*p*-Value	Gene-SNP Used in GRS Calculation	Minor Allele	Wildtype Reference Allele	Type of Effect
27.72 (9.24–46.21)	*p* = 0.003	ERCC2-rs50872	T	C	Main
		ERCC5-rs11069498	G	A	Main

**Table 4:** Impact of GRS on pain severity for women reporting pain: results of hurdle regression for the outcome model.

CME (95% CI)	*p*-Value	Gene SNP Used in GRS Calculation	Minor Allele	Wildtype Reference Allele	Type of Effect
0.45 (0.29–0.60)	*p* < 0.001	ERCC2-rs50872	T	C	Main
		ERCC5-rs11069498	G	A	Main
		ERCC5-rs4150360	C	T	Interaction
		ERCC5-rs4771436	G	T	Main
		PARP1-rs3219058	A	G	Interaction
		SEPP1-rs230819	A	C	Interaction

**Table 5:** Reported locations of pain at baseline and six months.

Pain Location	Baseline	Six Months
**Breast/axilla**	30	3
**Distal pain**	14	13
**No pain**	24	48

## Data Availability

De-identified data can be made available upon request (for research purposes) to qualified individuals within the scientific community.
